# Design of an 8‐Channel Transmit 32‐Channel Receive 11.7T Head Coil and Evaluation of SNR Gains

**DOI:** 10.1002/mrm.70473

**Published:** 2026-06-25

**Authors:** Son Chu, Jeslin Parackal Issac, Caroline Le Ster, Alexis Amadon, Vincent Gras, Paul‐François Gapais, Rüdiger Stirnberg, Nicolas Boulant, Shajan Gunamony

**Affiliations:** ^1^ Imaging Centre of Excellence University of Glasgow Glasgow UK; ^2^ MR CoilTech Limited Glasgow UK; ^3^ University of Paris‐Saclay, CEA, CNRS, BAOBAB, NeuroSpin Gif sur Yvette France; ^4^ MR Physics, German Center for Neurodegenerative Diseases (DZNE) Bonn Germany

**Keywords:** 11.7T, parallel transmit, receive array, transmit array, UHF, uiSNR

## Abstract

**Purpose:**

To design and develop an 8‐channel transmit 32‐channel receive 11.7T head coil for the strongest human MRI scanner to date, demonstrate safety, and evaluate signal‐to‐noise ratio (SNR) gains compared to 7T, the most used ultra‐high‐field (UHF) platform.

**Methods:**

The transmit array design incorporated a folded‐end radiofrequency (RF) shield to mitigate wave propagation in the scanner bore. The receive array consisted of 32‐overlapped loops constructed on a helmet size‐matched with the industry standard 7T head coil. The transmit array was characterized using B_1_^+^ mapping and MR thermometry, whereas the receive array performance was evaluated by measuring SNR at 7T and 11.7T using a lightbulb‐shaped phantom filled with a tissue equivalent solution.

**Results:**

Loss due to wave propagation was 4.5% of the input power for the chosen RF shield. The mean magnitude of the complex correlations between the simulated and measured single channel B_1_^+^ maps was 94%, which highlights minimal interaction between the transmit and receive arrays. An SNR gain of three‐fold was achieved in the phantom centre. In the periphery, representing the cortex, the SNR gain was 2.65‐fold, and the average gain over the whole volume was a factor of 2.69. The g‐factors of the 11.7T array were lower than the reference 7T coil due to the distinct coil sensitivities at higher frequency.

**Conclusion:**

We report substantial SNR gains over the whole volume at 11.7T using a fully overlapped 32‐channel loop array. The central SNR gain of B_0_^2.14^ agrees with the theoretical prediction, while we also gain supra‐linearly in the periphery.

## 1 Introduction

The theoretical supra‐linear gain in signal‐to‐noise ratio (SNR) with increasing static B_0_‐field is propelling the development of magnetic resonance imaging (MRI) systems toward 7T and beyond [1, 2, 3, 4, 5]. In 2001, the French Atomic Energy Commission launched the Iseult project, which aims to design an MRI magnet operating at 11.7T to investigate the human brain at mesoscopic resolution. After nearly 20 years of developing the 11.7T magnet, together with the scanner front and back‐end equipment, first images were acquired in 2021 in vitro [6].

To realize the benefits offered by the high static magnetic field and overcome the greater technical challenges at 11.7T, it is essential to develop unique and optimized instrumentation. This article presents the design and development of an 8‐channel transmit array in combination with a 32‐channel receive array developed for the Iseult 11.7T MRI scanner.

Radiofrequency (RF) coil design for MRI at 11.7T is challenging due to the design considerations associated with the Larmor frequency of 500 MHz for ^1^H imaging. It is well above the cut‐off frequency of a whole‐body ultra‐high field (UHF) MRI scanner bore, which is estimated to be about 300 MHz [7]. The conductive lining of the scanner bore acts as a waveguide and propagates the excitation signal applied to the transmit coil, resulting in a substantial loss in transmit efficiency. It was recently shown that this wave propagation can be mitigated by tailoring the structure of the local RF shield [8]. We demonstrated that an optimized RF shield combined with folded rings at the ends of the cylindrical shield confines the electromagnetic (EM) field within the RF coil and maximizes the transmit efficiency [8].

The B_1_^+^ field produced by the transmit coil is highly inhomogeneous because the RF wavelength in the human brain at 11.7T is only 7 cm—about one‐third of the human head's largest dimension—and the constructive and destructive interferences can cause multiple signal maxima and minima within the imaging volume. The inhomogeneous EM field results in a spatially dependent energy deposition, increasing the potential for localized RF heating. The RF power absorbed per unit mass is controlled by the specific absorption rate (SAR) limits during routine examination. Therefore, the ability to provide an efficient and homogeneous RF excitation while operating within the SAR constraints is one of the most important engineering requirements in designing RF transmit coils for MRI at 11.7T.

Parallel imaging techniques [9, 10, 11] and UHF are complementary due to the distinct coil sensitivities caused by the higher resonance frequency and reduced wavelength [12, 13]. To take advantage of the increased SNR offered by the higher magnetic field and to benefit from the improved parallel imaging performance, UHF transmit arrays are combined with receive arrays constructed on close‐fitting helmets. A variety of UHF receive arrays ranging from 32 up to 128 receive elements have been developed [1, 13, 14, 15, 16, 17, 18, 19]. This includes conventional loop arrays, combinations of loops and dipoles, as well as combinations of receive and transceiver arrays with an aim to maximize the SNR achieved with respect to the ultimate intrinsic SNR (uiSNR)—the theoretically highest possible SNR for a given sample [20, 21, 22].

Engineering and implementation of UHF arrays are challenging due to the strong interactions between the transmit and receive arrays, which can invalidate the SAR evaluations. In this paper, we characterize both the transmit and receive performance of the 11.7T coil. Transmit array performance was compared with the EM model, and RF safety was validated using established methods [23, 24, 25]. The SNR gains and parallel imaging performance at 11.7T were compared with the industry standard 32‐channel 7T head coil.

## 2 Methods

### 2.1 EM Simulations

The transmit array design followed the workflow presented in our earlier work, which combined EM and RF pulse design (RFPD) simulations to choose the optimum transmit array [8]. EM simulations were performed using the time‐domain solver in CST Studio Suite 2024 (Dassault Systèmes, France), which employs the finite‐integration technique along with RF circuit co‐simulation [26, 27]. An 8‐channel transmit array was chosen to match the number of transmit channels in the scanner. We opted for conventional loop transmit elements due to their robustness across a wider range of loading conditions compared to distributed resonant structures such as dipoles [28, 29, 30].

Each loop consisted of 18 evenly distributed capacitors (17‐fixed and one variable) to tune to 499.415 MHz. The capacitors were connected using 2‐mm diameter perfect electric conductor (PEC) wires. The array elements were arranged on a fiberglass tube with an inner diameter of 290 mm and a wall thickness of 2.5 mm, with electrical properties *ε*_r_ = 5.5 and tanδ=0.04. The loop length was set to 185 mm along the z‐direction. The numerical model also incorporated the coil housing, scanner bore, and components, as well as dielectric losses and coil cable loss until the coil plug. Each loop was matched to a head‐and‐shoulders phantom (*ε*_r_ = 48.73, *σ* = 0.65 S/m) [8] using a balanced matching circuit [31]. Variable capacitors for tuning and matching were adjusted in circuit co‐simulations to achieve an impedance match of better than −40 dB in each channel. The receive array was not included in the numerical model; however, the helmet, including the EM properties of its material, was included to accurately position the phantom and body models.

**FIGURE 1:**
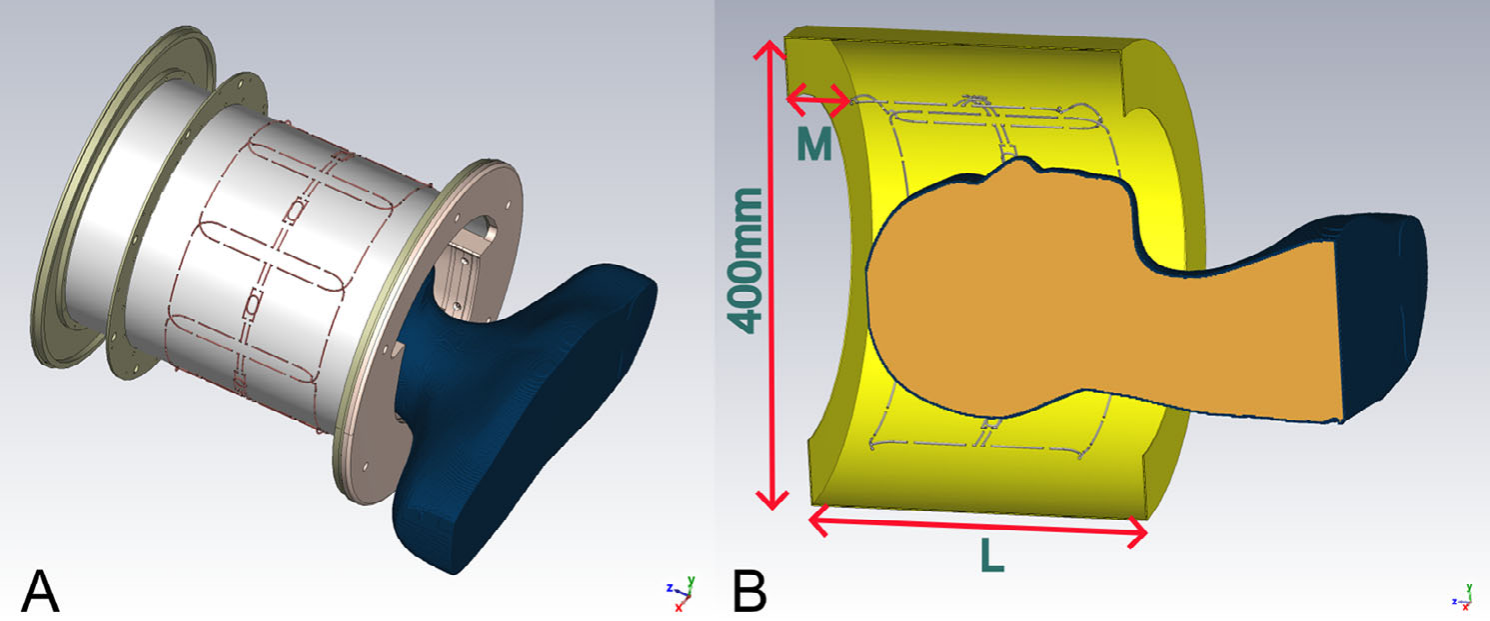
(A) Numerical model of the transmit array in CST, where the adjacent elements are decoupled by geometric overlaps and the next‐neighboring elements are decoupled by nested overlaps. (B) Cross‐sectional view of the RF shield, comprising a cylindrical section with folded‐end rings at both ends. The distance between the ends of the loop elements and the folded ends of the RF shield (M) was varied to optimize the length of the RF shield (L).

There was strong coupling between the next‐neighboring elements due to the large size of the loops. A nested layout (Figure 1A) was used to geometrically decouple the adjacent elements, and an extension through the middle was created to overlap and decouple the next‐neighboring channels [32]. The RF shield was optimized to mitigate wave propagation. The shield consisted of a cylindrical section and folded‐end rings, one at each end of the cylinder (Figure 1B). We limited the diameter of the cylinder to 40 cm to align the coil height close to the isocentre and to manage the overall size of the coil, although marginal gains in transmit efficiency were expected with a larger diameter [33]. The length of the RF shield was optimized by varying the distance between the ends of the loop and the folded ends [8].

Local mesh refinement was applied with a step size of 0.7 mm in all directions to the electric conductors. Broadband signal excitations from 440 to 540 MHz were used with perfectly matched layers placed at a distance of one‐fourth of the wavelength at 499.415 MHz. The accuracy of numerical simulations was secured by setting a convergence criterion of −40 dB for the amplitude of the port signals at the end of the simulation time interval. A typical simulation consisted of ∼74 million mesh cells and took ∼25 h on a customized Z8 G4 HP workstation equipped with a dual Xeon Gold 5222 processor, 96GB RAM, and one NVidia Quadro RTX 5000 GPU acceleration card.

The simulated EM fields from CST were exported on a 5‐mm isotropic grid and used as the input for RFPD simulations. These simulations, which aimed to homogenize the flip angle within a brain mask, were performed under realistic constraints to guide the design of the transmit array (loop dimensions) and the RF shield. Further details about the RFPD simulations can be found in Reference [8].

### 2.2 Transmit Array Construction

**FIGURE 2:**
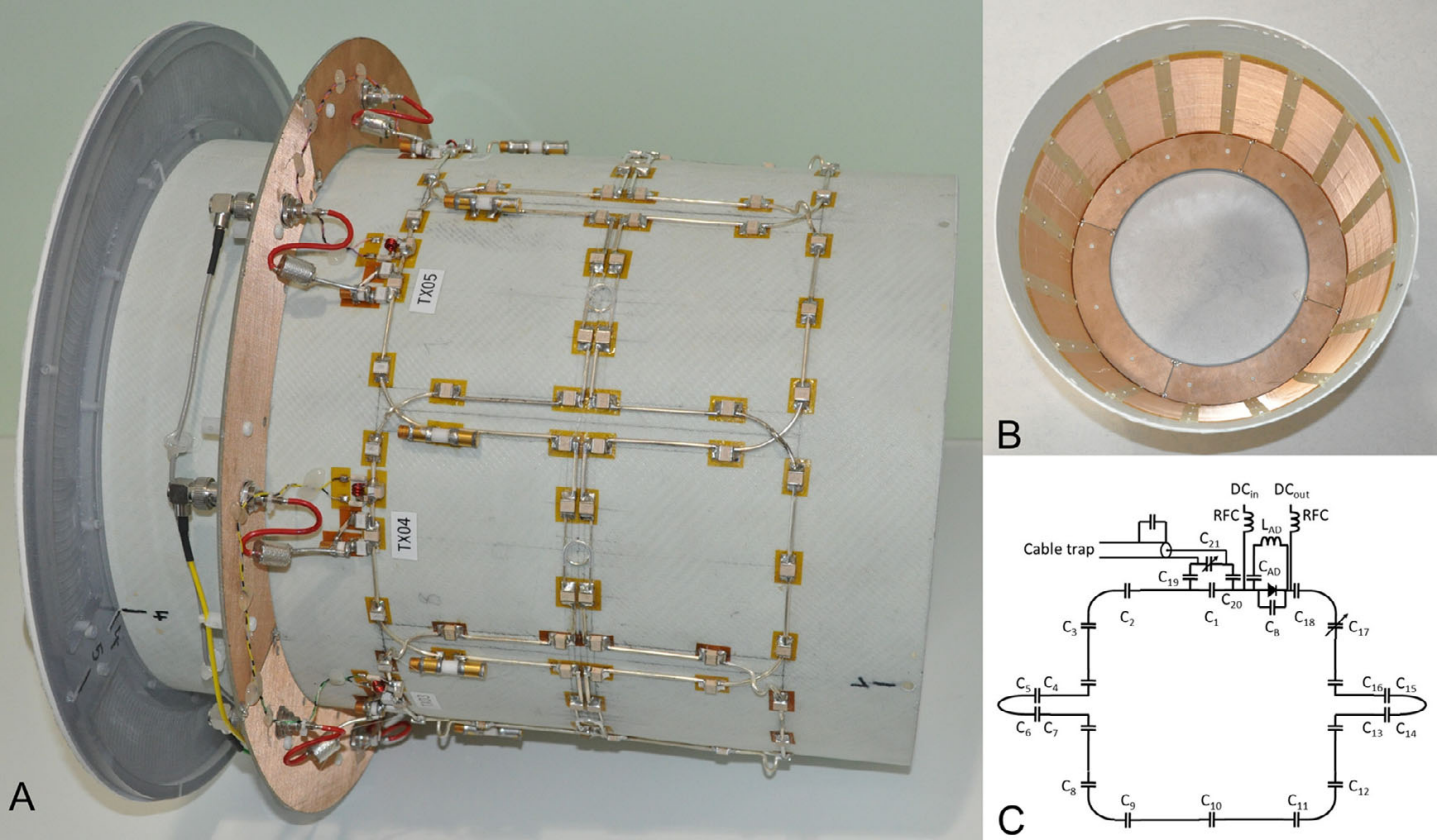
Photograph of (A) the constructed 8‐channel transmit array and (B) the implementation of the folded‐end PBM RF shield with 16 strips. (C) Equivalent circuit diagram of a single transmit element, featuring all loop components and the active detuning circuitry.

The constructed transmit array is shown in Figure 2A. There were two concentric fiberglass tubes with inner diameters of 290 mm and 400 mm held together using two end plates, one at each end. The RF shield was realized using fine phosphor bronze mesh (PBM) (380 mesh/in., Shandong Xingying Technology, China). We showed in a recent study that PBM substantially minimizes acoustic noise caused by the induced eddy currents in the RF shield while preserving transmit efficiency [34]. Sixteen PBM strips were attached to the inner surface of the outer tube with a gap of 2 mm between the strips. For RF continuity, three 390‐pF capacitors were evenly spaced and soldered between adjacent strips. The folded‐end sections of the RF shield were realized as shown in Figure 2A,B. The folded sections of the RF shield were implemented on the inner surfaces of the lower end plate and a middle ring, effectively forming two annular sections. There were four segments in the folded end, also separated by 2‐mm gaps and bridged with 390‐pF capacitors.

To construct the transmit loops, solder pads were attached to the outer surface of the inner tube. Each loop consisted of 17x 2.4‐pF capacitors (800C‐series; KYOCERA AVX Components Corporation, USA) and a variable capacitor (1–7.5 pF, 5610, Johanson Manufacturing Corporation, USA). The capacitors were connected using 2‐mm diameter silver‐plated copper wire (Scientific Wire Company, UK).

The active detuning circuit for each coil element consisted of a PIN diode (MA4P7446F‐1091T, MACOM, USA) connected in series. A series LC circuit was connected in parallel to the diode to tune out the residual capacitance and improve isolation at the Larmor frequency when the diode was turned OFF. The equivalent circuit of a single transmit element is shown in Figure 2C. A shielded two‐turn cable trap tuned to 499.415 MHz was placed between the coil input and the system cable. It provided −30 dB isolation on average. The construction process of the cable trap is explained in References [35, 36].

### 2.3 Receive Array Construction

The receive array consisted of 32 elements symmetrically arranged in four rows on the surface of an anatomically shaped helmet with internal dimensions (left/right: 186 mm; anterior/posterior: 220 mm) matching the industry standard 7T head coil (Nova Medical Inc., USA). There were nine channels in each of the top three rows, and the remaining five elements were on the fourth row. Each element geometrically overlapped with the adjacent loop in each row and with two elements in the lower row.

**FIGURE 3:**
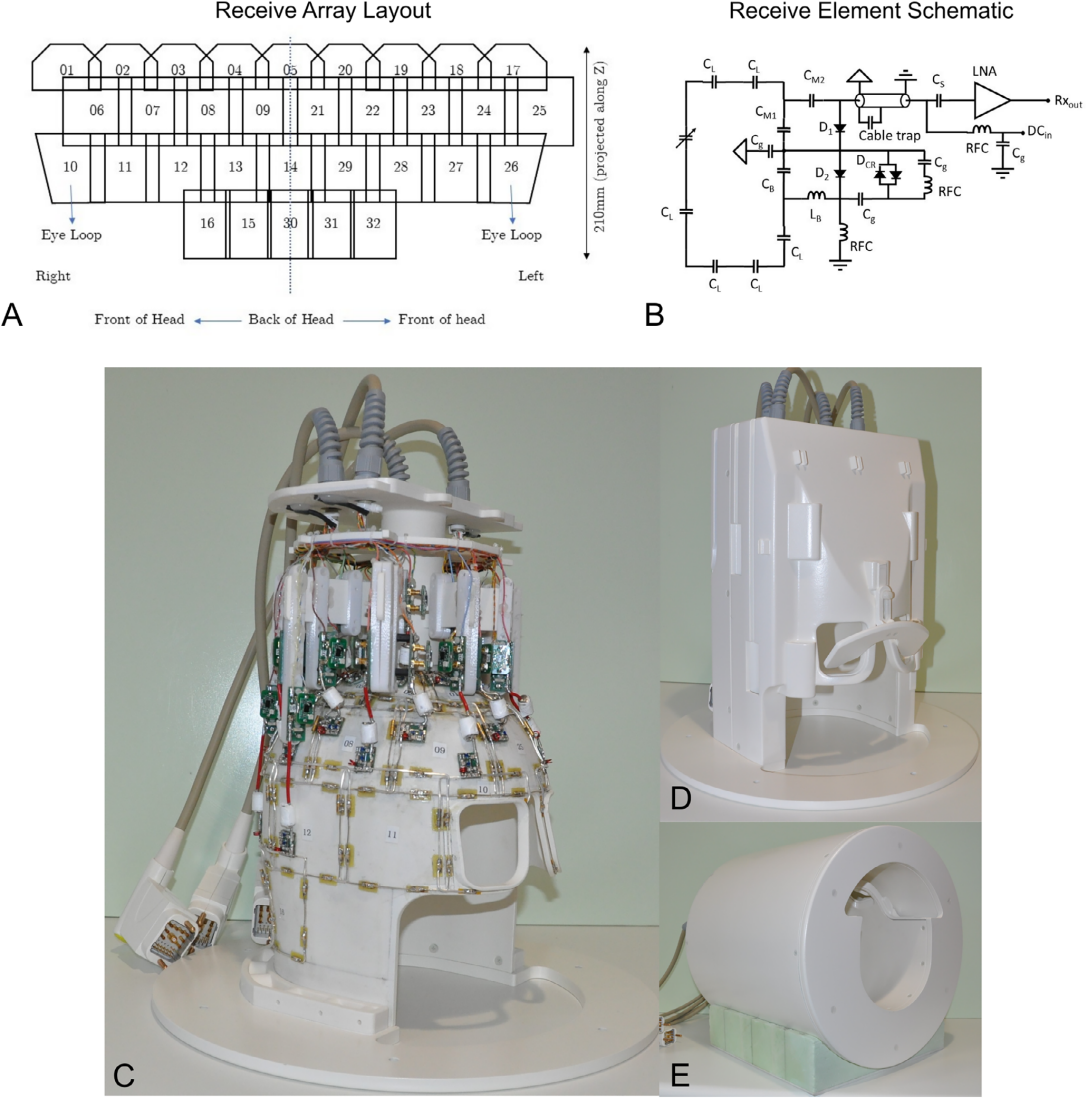
(A) 2D layout of the receive array, illustrating the symmetric arrangement of 32 receive elements. (B) Equivalent circuit diagram of a single receive element. Photograph of the constructed receive array (C) without and (D) with enclosure, which includes built‐in pockets for future integration of B_0_‐field monitoring probes. (E) Photograph of the fully assembled coil. There is a rear projection mirror in the space between the transmit and receive arrays.

To help with coil construction, grid lines were drawn on the surface of the helmet, consisting of 4 equally spaced horizontal lines to mark the boundary of each row. Each horizontal line is segmented equally to form nine segments to mark the grid line for each element. The vertical lines in the adjacent rows bisected the horizontal lines of the neighboring row. The loops are built and overlapped by using the grid lines as reference. The 2D layout of this arrangement is shown in Figure 3A. The dimensions of the grid in each row are shown in Figure S1.

The top row elements covering the dome of the helmet were smaller in size and required a total of six evenly distributed capacitors (5‐fixed, 1‐variable), whereas all the other loops were tuned to 499.415 MHz with eight capacitors (7‐fixed, 1‐variable). One of the fixed capacitors was replaced with an input board, which consisted of a matching circuit, an active detuning circuit, and a passive detuning circuit. The equivalent circuit is shown in Figure 3B, and the components are listed in Table S1. All capacitors in the resonant loop were evenly spaced and assembled on solder pads glued on the helmet surface and connected using 1.25‐mm diameter solid “dead soft” 99.99% fine silver wire (Cookson Precious Metals, UK). Our aim was to minimize electronics losses wherever possible at the high operating frequency of 11.7T, which resulted in the choice of this material. The silver wires provided 10% higher unloaded Q compared to the commonly used silver‐plated copper wire (SPCW). In addition, constructing the coil and adjusting the overlaps with the soft silver wire is easier compared to SPCW.

Each loop was connected to a low input impedance preamplifier (WMA500D, WanTcom Inc., USA), and preamplifier decoupling was adjusted by varying the series capacitor (C_s_) between the coil input and preamplifier. The active detuning circuit consisted of two PIN diodes in series, with the first PIN diode (D_1_) creating a short across the input capacitors (C_M1_ and C_M2_) and the second PIN diode (D_2_) creating a high impedance across the blocking capacitor (C_B_) when turned ON. A shielded two‐turn cable trap [35, 36] was connected between the coil input and the preamplifier. To minimize interactions between the transmit and receive arrays, receive electronics and cabling were lifted above the level of the transmit elements, and those within the transmit field were routed along the virtual ground of the transmit loops.

A photograph of the fully assembled receive array is shown in Figure 3C. To protect the electronics from ingress, the completed receive array was enclosed as shown in Figure 3D. The receive array housing was 3D printed using selective laser sintering technology with flame retardant PA2210FR polyamide powder (Ogle Models, UK). The external surface of the coil housing was finished with Siemens recommended biocompatible paint. The transmit and receive arrays were tuned, matched, and decoupled in the lab by loading with a head and shoulder phantom [8, 14]. Custom‐built test rigs that provide control signals to mimic the scanner interface were used to characterize the arrays in the lab. A photograph of the completed coil is shown in Figure 3E.

### 2.4 RF Safety Validation

To ensure coil safety as well as compliance with SAR guidelines from IEC, we followed the established workflow for home‐built coils at Neurospin, CEA, and the methods and recommendations provided in References [23, 24, 25]. EM simulations were performed with two body models (Duke and Ella) from the Virtual Family cohort [37] for three head positions in the coil. In position 1, the body model is inserted into the helmet to the maximum in the head direction. The body models are moved toward feet by 10 mm in position 2, and in position 3 the models are lifted in the Y‐direction by 5 mm from position 1. From each electric field simulation, 10‐g averaged local SAR matrices were computed. The SAR matrices were then pooled together and the obtained set was compressed using an iterative approach [38] to form the virtual observation points (VOPs) [39], which is the local SAR prediction model for this coil. The VOP file generated for SAR management incorporated a simulation error propagation term following the method recently proposed in Reference [40] (equation 14) and an intersubject variability factor of 1.3 derived from the six body model simulations.

Maximum voltage per channel was set to 240 V at the coil plug. SAR limits were 3.2 and 20 W/kg for global and local SAR, respectively. To compare the measured B_1_^+^‐field maps with the numerical model, an agar gel phantom (diameter 15.6 cm; 25 g/L agar, 4 g/L NaCl, 0.25 g/L CuSO_4_; *ε*_r_ = 76.6, *σ* = 0.83 S/m) was prepared. This phantom was simulated, and its position was matched between simulation and measurement within ±2 mm. B_1_^+^‐field maps were acquired using a Turbo FLASH sequence (TR/TE = 15 000/1.73 ms, 40 slices, 2.5 mm thickness, FOV = 320 mm, matrix = 64, reference amplitude = 360 V, bandwidth = 1500 Hz/px, turbo factor = 44). The simulations were compared to the measurements to determine a calibration matrix [Lc_ij_] by fitting the measured field maps with a linear combination of the simulated ones, expressed as $B_{1,k}^{\text{meas}}≅B_{1,k}^{\mathrm{cal}}=\sum_{n=1}^{N}{\mathrm{Lc}}_{k,n}B_{1,n}^{\mathrm{sim}}$, where N is the number of channels [25]. The calibration matrix was computed iteratively via least‐squares fits, enabling redefinition of the simulations ($B_{1,n}^{\mathrm{sim}}\to B_{1,k}^{\mathrm{cal}}$) to correct for unaccounted losses (e.g., solder joints, lumped elements) and coupling between channels [25, 41].

The calibration matrix thereby yielded a new set of simulated E‐field maps for the phantom, which were verified experimentally with MR thermometry using circularly polarized (CP) mode with the same phantom. The voxel‐wise SAR values (SAR = σ*E^2^*dutycycle/(2*ρ)) were calculated and integrated in the heat diffusion equation. MR thermometry experiments were performed using a 3D GRE sequence (TE = 20 ms, 60 slices, 3 mm thickness, FOV = 192 mm, matrix = 64, RF amplitude = 100 V, nonselective square excitation, 1‐ms duration). RF heating in the phantom was obtained by acquiring two such sequences separated by 6 min of an RF‐only sequence, with no gradient activity to prevent heating of the iron passive shim and minimize field drifts. The scanner field drift was characterized prior to the thermometry measurements by looking at the B_0_ field map differences over 6 min without any activity. TR was set to 50 ms, which led to approximately 4 W per transmit channel at the RF amplifier output. The phantom was left overnight in the scanner room before the scan, so that the phantom temperature was equal to the room temperature at the beginning of the scan.

Measured temperature maps (ΔT) were computed from phase differences (Δϕ) using ΔT=Δϕ/(2πανTE), with α = −0.01 ppm/°C, ν = 500 MHz, and TE = 20 ms. The simulated temperatures were computed with *σ* = 0.83 S/m, *ρ* = 1000 kg/m^3^, thermal conductivity *k* = 0.5 W/m/K, Cp = 4000 J/kg/K. Once the calibration matrix was determined and the comparisons between the B_1_^+^‐field and temperature measurements on the phantom were deemed satisfactory, the same calibration matrix was applied to the simulated E‐field maps computed on the different head models.

### 2.5 SNR and g‐Factor Mapping

The receive performance of the constructed 11.7T 32‐channel array was compared with the most widely used 32‐channel 7T head coil (1Tx32Rx 7T head coil, Nova Medicals Inc., USA). A lightbulb‐shaped phantom [18] was 3D printed, and the phantom solution was prepared using polyvinylpyrrolidone (PVP10) and sodium chloride. Further details of the phantom can be found in Supporting information.

Images were acquired using the same phantom at both 7T and 11.7T, and the position of the phantom in the receive array was matched by using the same padding. To quantify SNR at both field strengths, we used two identical 3D GRE protocols with a nominal 5° flip angle (resolution = (2.5 mm)^3^, TR = 300 ms, bandwidth = 500 Hz/pixel, TE = 5 ms, matrix size = 100 × 78 × 80). A noise scan with 0 V and identical parameters except for TR = 50 ms was acquired to generate the noise covariance matrix (Ψ) at each field strength. The number of samples to compute the covariance matrix was 1 248 000. Following T_1_ estimation via three GRE acquisitions at varying flip angles, the flip angle was measured for each mode of excitation using an AFI sequence [42] (identical resolution; TR = 300 ms, TR_2_/TR_1_ = 5) to correct for flip‐angle inhomogeneity via the GRE signal equation, thereby isolating contributions from the receive array. The corrected SNR was then computed as $\mathrm{SNR}=\sqrt{S^{\prime }\Psi^{-1}S}$, where S is the 32 × 1 signal vector from the receive channels [43]. The TE of the AFI and 3D GRE acquisitions was set to 5 ms to avoid spurious oscillations due to PVP bands found in 1H‐NMR spectra [44]. System gain settings were identical at both field strengths. In addition, scanner room temperatures were controlled and monitored to be within 18°C–20°C [45].

To improve accuracy in the very low flip angle regions at 11.7T, the GRE and AFI sequences were run twice with complementary RF shim modes (CP and CP2+ modes). Following flip angle correction for each mode, the final SNR map was computed as a weighted average of the mode‐dependent SNR maps, with signal acting as the weight in each voxel. At 7T, SNR was characterized only in CP mode.

The SNR gain achieved at 11.7T compared to the industry standard reference 7T coil was quantified in the central and cortical regions, and in the whole volume of the lightbulb phantom. The central region of interest (ROI) was defined as a 15‐mm‐diameter sphere, and the cortical ROI was made of a 20‐mm thick layer along the edge of the phantom. The rest of the phantom comprised all remaining internal volumes not assigned to the central, cortical, or uncalculated regions, whereas the whole‐brain volume was defined as the sum of the central, cortical, and rest‐of‐phantom regions (Figure 8B). SNR gain was computed as a ratio of each voxel within the ROI and averaged for each region.

Geometrical factor (g‐factor) maps were computed from the GRE acquisitions using the SENSE reconstruction framework with individual receive channel SNR maps acting as coil sensitivity maps (per equation (23) in Reference [10]). In this g‐factor evaluation, we used Pruesmann's formula without regularization nor coil compression [10].

### 2.6 In Vivo Imaging

Approval to scan healthy volunteers using the coil has been granted by the regulatory body and ethics committee. In vivo images acquired with one volunteer, who had signed an informed consent, are reported here. All acquisitions made use of home‐made RF pulse design algorithms to mitigate B_1_^+^ field inhomogeneities. The 3D‐EPI sequences also incorporated prospective motion correction and correction of first‐order field changes [46].

*Anatomical 3D‐EPI slab‐select acquisition, 0.3 mm resolution*: Resolution = 0.3 × 0.3 × 0.3 mm^3^, matrix = 484 × 660 × 400, axial orientation, FA = 13° (time bandwidth product = 15), TE = 17.5 ms, TR = 43 ms, iPAT = 2 × 2, echo spacing = 2.34 ms, BW = 516 Hz/px, slice oversampling = 7.5%, EPI factor = 10, 7/8 phase PF, segmentation = 30, TA = 5 min.

*Anatomical 3D‐EPI slab‐select acquisition, 0.2 mm resolution*: Resolution = 0.2 × 0.2 × 0.2 mm^3^, matrix = 684 × 932 × 600, axial orientation, FA = 13° (time bandwidth product = 15), TE = 17.5 ms, TR = 45 ms, iPAT = 2 × 2, echo spacing = 3.34 ms, BW = 336 Hz/px, slice oversampling = 6.7%, EPI factor = 7, 6/8 phase PF, segmentation = 50, TA = 2 × 12.5 min = 25 min (two reversed polarity acquisitions were averaged for compensation of eddy‐currents). A minimum intensity projection along the z axis (3 mm thickness) was also computed from this data to highlight vasculature.

*MP2RAGE*: Resolution = 0.6 × 0.6 × 0.6 mm^3^, matrix = 318 × 338 × 240, sagittal orientation, TR = 5.1 s, iPAT = 4, TI1/TI2 = 991/2970 ms, FA1/FA2 = 4/3 deg., TA = 10 min 20 s.

## 3 Results

### 3.1 Transmit Array

**FIGURE 4:**
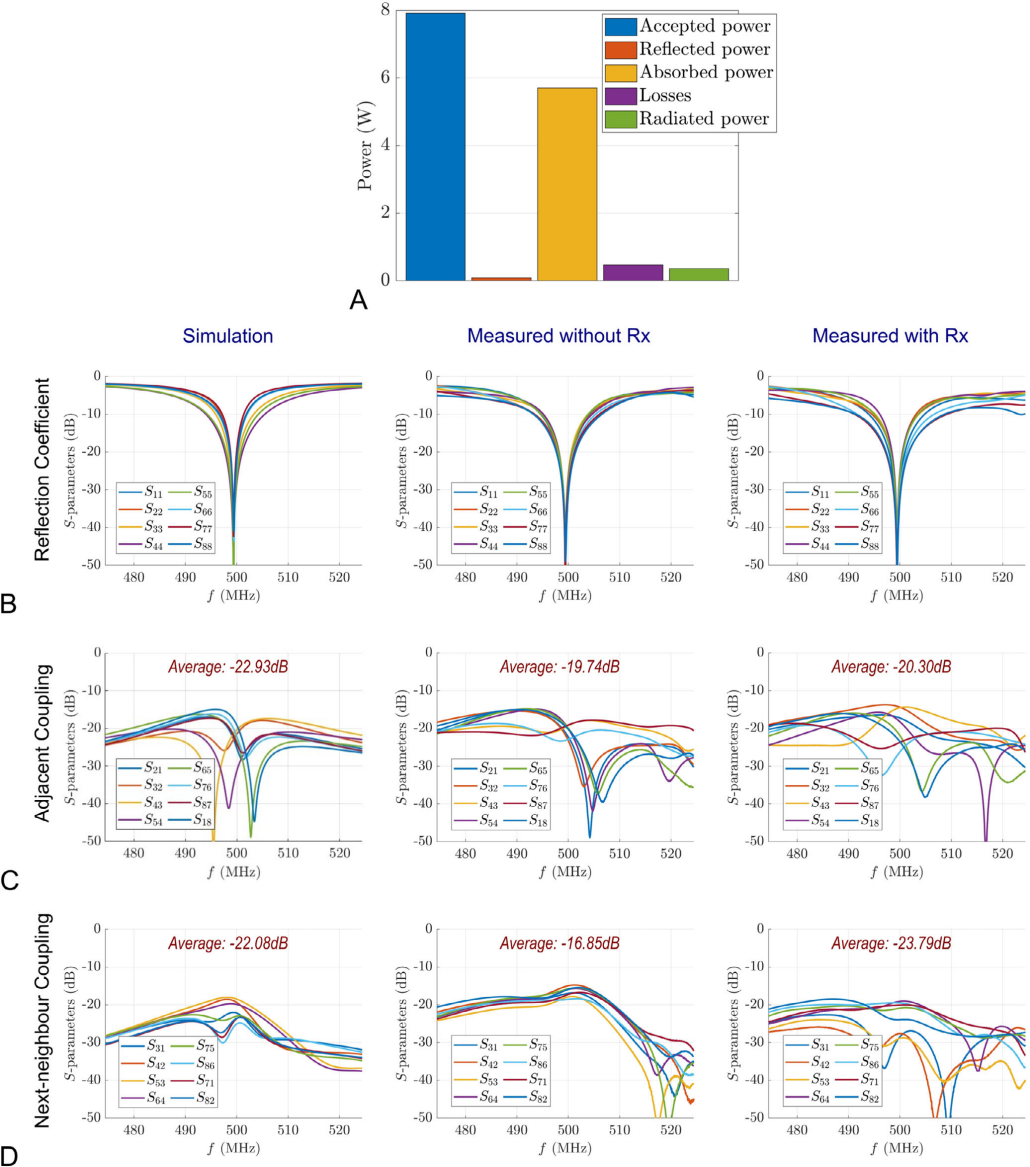
(A) Simulated power budget of the transmit array loaded with a head‐and‐shoulder phantom for 8 W input power in CP mode. The transmit array S‐parameter plots are shown in (B, C and D). Column 1: Simulated; column 2: Without receive array and column 3: With receive array. The reflection coefficient plots are shown in (B), the coupling to the adjacent elements is shown in (C) and the coupling to the next‐neighboring channels is shown in (D).

The simulated power budget for the finalized transmit array is shown in Figure 4A. The chosen RF shield had a diameter of 40 cm, and the distance from the folded‐end to the edge of the loop was 5 cm. Accepted power, which is the difference between the input power and the reflected power due to coupling and mismatch, was about 99% of the input power. This was due to the very low reflected power achieved by the excellent decoupling performance with the nested layout, which decoupled both the adjacent and next‐neighboring channels. The loss due to radiation was 4.5% of the input power. It is however possible to minimize the radiation loss further by increasing the diameter of the RF shield [8, 33, 47]. We opted to restrict the RF shield diameter to 40 cm to manage the overall size of the coil and the height of the coil on the patient table. Dielectric lining and additional annular rings can alter the dispersion characteristics of the RF shield and minimize radiation loss [48], which will be part of future studies.

Resistive losses accounted for about 6% loss in the power budget. This was largely due to the series resistance of the multiple capacitors essential to tune the loops to 500 MHz. In this design, 800C series capacitors, which offer the least ESR among the C‐series capacitor range, were used. Further reduction would be possible by choosing components with lower ESR.

The measured S‐parameters of the transmit array with and without the actively detuned receive array are shown in Figure 4B–D. Only the tune and match of the transmit loops were adjusted after inserting the receive array. All eight channels were matched to better than −40 dB (Figure 4B). Clear resonance can be seen on all the reflection coefficient plots with no evidence of coupling between the transmit elements or interaction between the transmit and receive arrays. The decoupling between the adjacent elements was close to −20 dB (Figure 4C). The presence of the receive array further damped the coupling between the next neighboring channels to less than −20 dB on average (Figure 4D). The S‐matrix of the transmit array at 499.415 MHz is shown in Figure S5. The S_21_ values, measured with a pair of decoupled flux probes when the PIN diode was turned OFF, were less than −60 dB on average.

### 3.2 Receive Array

The unloaded Q‐factor of a single receive loop, measured in isolation by placing it inside a cylindrical PBM RF shield, was 144 at 500 MHz. The loaded Q‐factor varied from 18 to 57 for a separation from 5 mm to 25 mm between the loop and the lightbulb phantom. The separation was measured between the inner wall of the helmet and the outer wall of the phantom. The Q‐ratio between 2.5 and 8 demonstrated sample noise dominance under lightly to heavily loaded conditions.

While constructing the receive array, the helmet was securely placed over the head and shoulder phantom on the lab table, which allowed all adjustments to be made in the loaded condition. Each receive element was matched to better than −20 dB, and decoupling between the adjacent elements within the row was at least −10 dB, while it was lower than −15 dB between the adjacent elements within two rows. In addition, preamplifier decoupling was lower than −20 dB on average. This is the difference between the S_21_ values measured with a pair of decoupled flux probes when the loop was terminated with a 50‐Ω load and the low impedance preamplifier. The active detuning measured similarly by biasing the PIN diodes through the test rigs averaged lower than −30 dB.

### 3.3 Transmit Array Characterization

**FIGURE 5:**
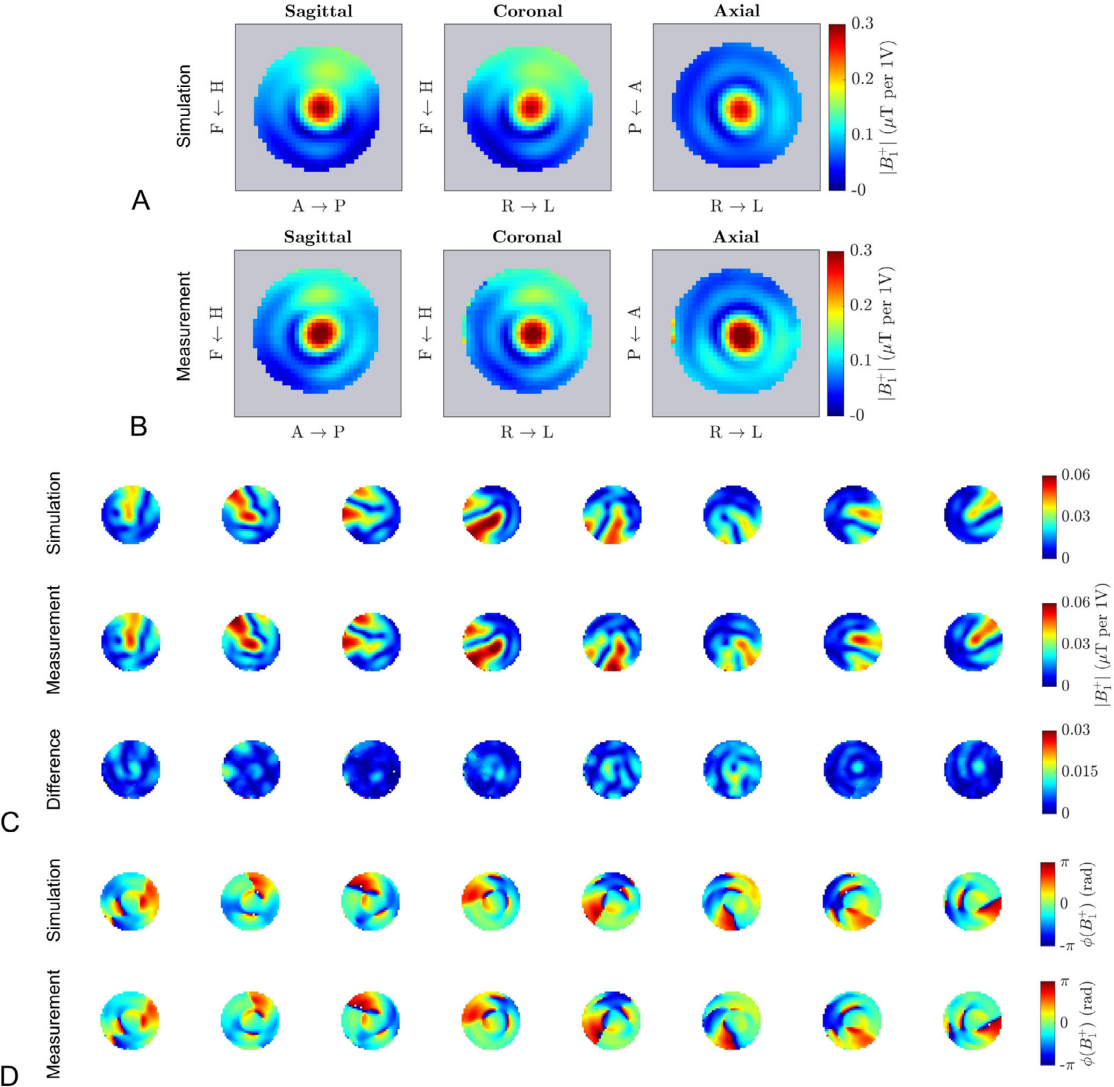
(A) Simulated (with calibration) and (B) measured B_1_^+^‐field map in the spherical agar phantom in CP mode for 1‐V excitation per channel at the output of the RF amplifier. (C) Simulated and measured single channel B_1_^+^ magnitude maps and the difference maps. (D) Phase maps of the central axial slice. Each channel was excited with 1‐V excitation while the remaining channels were terminated to 50 Ω.

To validate RF safety, circular polarized (CP) mode and single channel B_1_^+^‐field maps were acquired using the spherical agar phantom. Figure 5 presents the simulated (A) and measured (B) CP mode field map for 1‐V excitation per channel at the output of the RF amplifier. Simulated and measured single channel magnitude and phase maps of the central axial slice are shown in Figure 5C,D.

**FIGURE 6:**
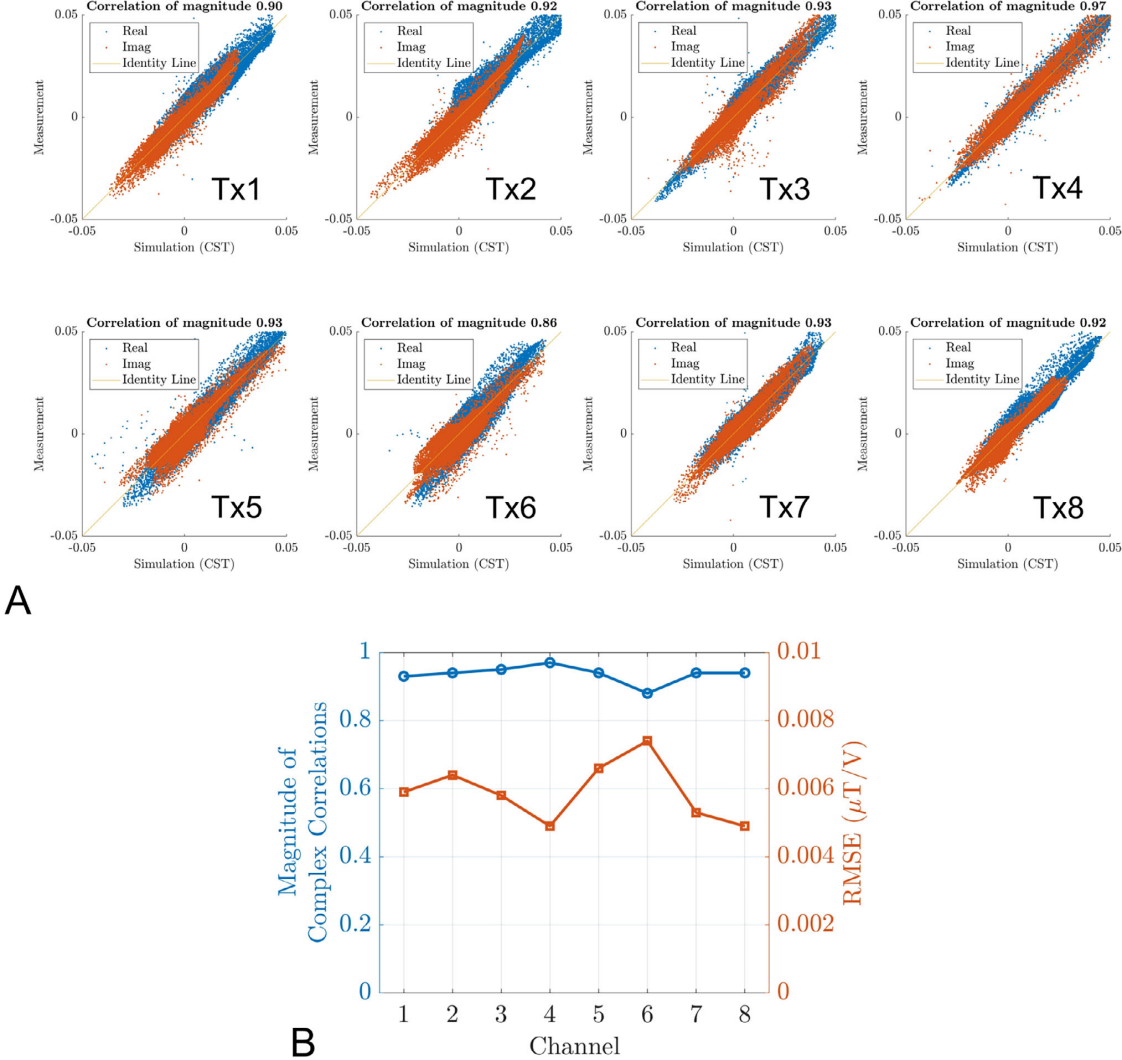
(A) Scatter plots of measured versus calibrated simulated B_1_^+^ values for all transmit channels (real and imaginary components shown in different colors). (B) The magnitude of complex correlations and the RMSE between measured versus calibrated simulated B_1_^+^ values for each transmit channel.

The agreement between the calibrated B_1_^+^‐field simulations and measurements is quantified using the scatter plots for each transmit channel, as shown in Figure 6A. The plots show the corresponding simulated and measured values for each voxel in space, and their correlation provides a measure of the agreement between the two. The magnitude of the complex correlations and the RMSE values are shown in Figure 6B. The mean magnitude of the complex correlation and the RMSE was 0.94 and 0.0059, respectively, indicating minimal interaction between the transmit and receive arrays.

**FIGURE 7:**
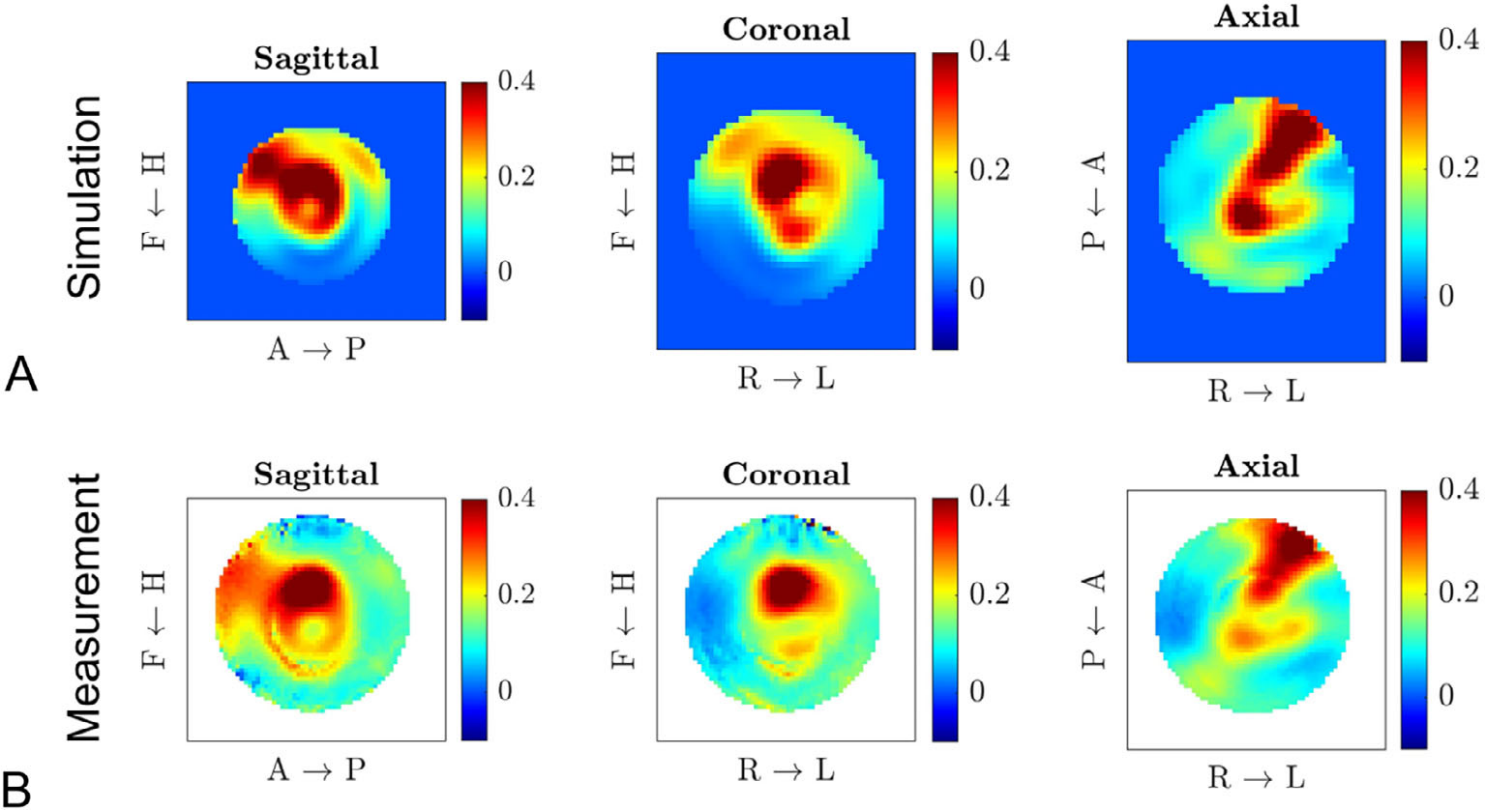
MR thermometry experiments with the CP RF excitation mode. (A) Simulated and (B) measured temperature rise (°C) for the CP mode with 4 W per channel, applied for 6 min of RF heating.

The simulated (A) and measured (B) temperature maps for the CP mode are shown in Figure 7. The spatial distribution of the measured heating pattern reproduced to a large extent the simulated heating patterns. Furthermore, the hot spots also appeared to match quantitatively. A discrepancy of approximately 0.1°C was observed between the measurements and the simulations and was homogeneous over the phantom, suggesting possibly small and residual unaccounted field drifts.

### 3.4 SNR Comparison

**FIGURE 8:**
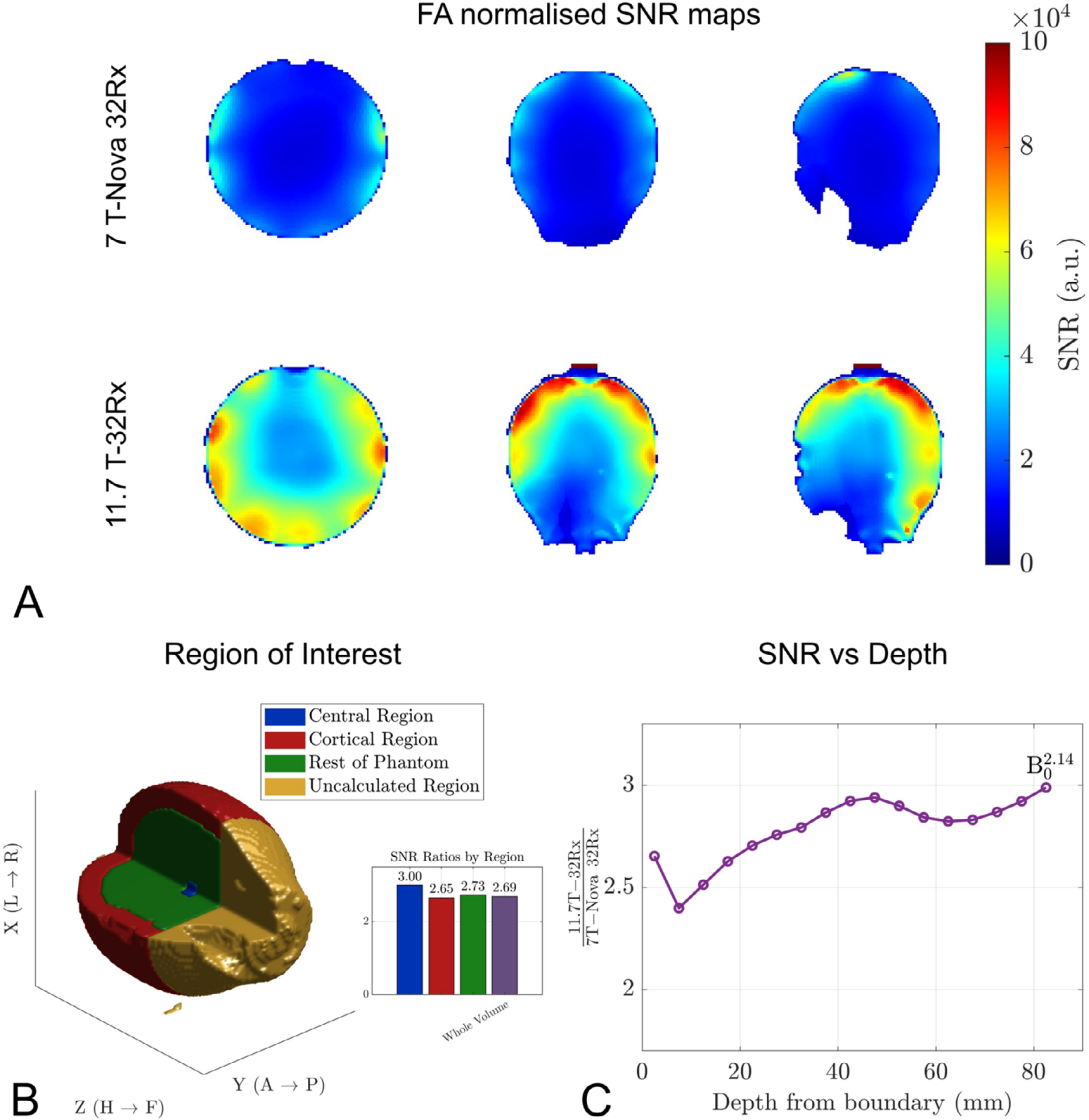
(A) SNR maps from three orthogonal slices of the lightbulb‐shaped phantom acquired with the 32‐channel NOVA coil at 7T (top) and the 32‐channel custom‐built coil at 11.7T (bottom). (B) Phantom compartment structure used for analysis, showing an overall SNR gain of 2.69‐fold across the entire brain region, with gains of 3.0‐fold, 2.65‐fold, and 2.73‐fold in the central, cortical, and remaining regions, respectively. (C) SNR ratio as a function of depth, averaged over 5 mm‐thick concentric shells.

Flip‐angle normalized 7T and 11.7T SNR maps for the lightbulb phantom are shown in Figure 8A. The different regions of the phantom considered for the comparison are shown in Figure 8B. Statistical significance was assessed using a paired *t*‐test on the voxel‐wise SNR ratios within each ROI. In the central 15‐mm spherical ROI (blue), the SNR gain was a factor of 3 (mean = 2.9968; standard deviation = 0.0594; *p*‐value: < 0.0001). In the 20‐mm peripheral ROI (red) representing the cortex, the SNR gain was a factor of 2.65 (mean = 2.6517; standard deviation = 1.1897; *p*‐value = 0.0258). The region represented in green gained a factor of 2.73 (mean = 2.7272; standard deviation = 0.4557; *p*‐value < 0.0001), and the average gain in the whole volume of the phantom was a factor of 2.69 (mean = 2.6909; standard deviation = 0.8899; *p*‐value = 0.0025), demonstrating substantial gains over the entire volume with the 32‐channel overlapped loop array at 11.7T. Figure 8C depicts the SNR gain as a function of depth, derived from SNR values averaged across 5 mm‐thick concentric shells in the central axial slice, showing consistent supra‐linear gains across the whole phantom, from the surface to the centre.

**FIGURE 9:**
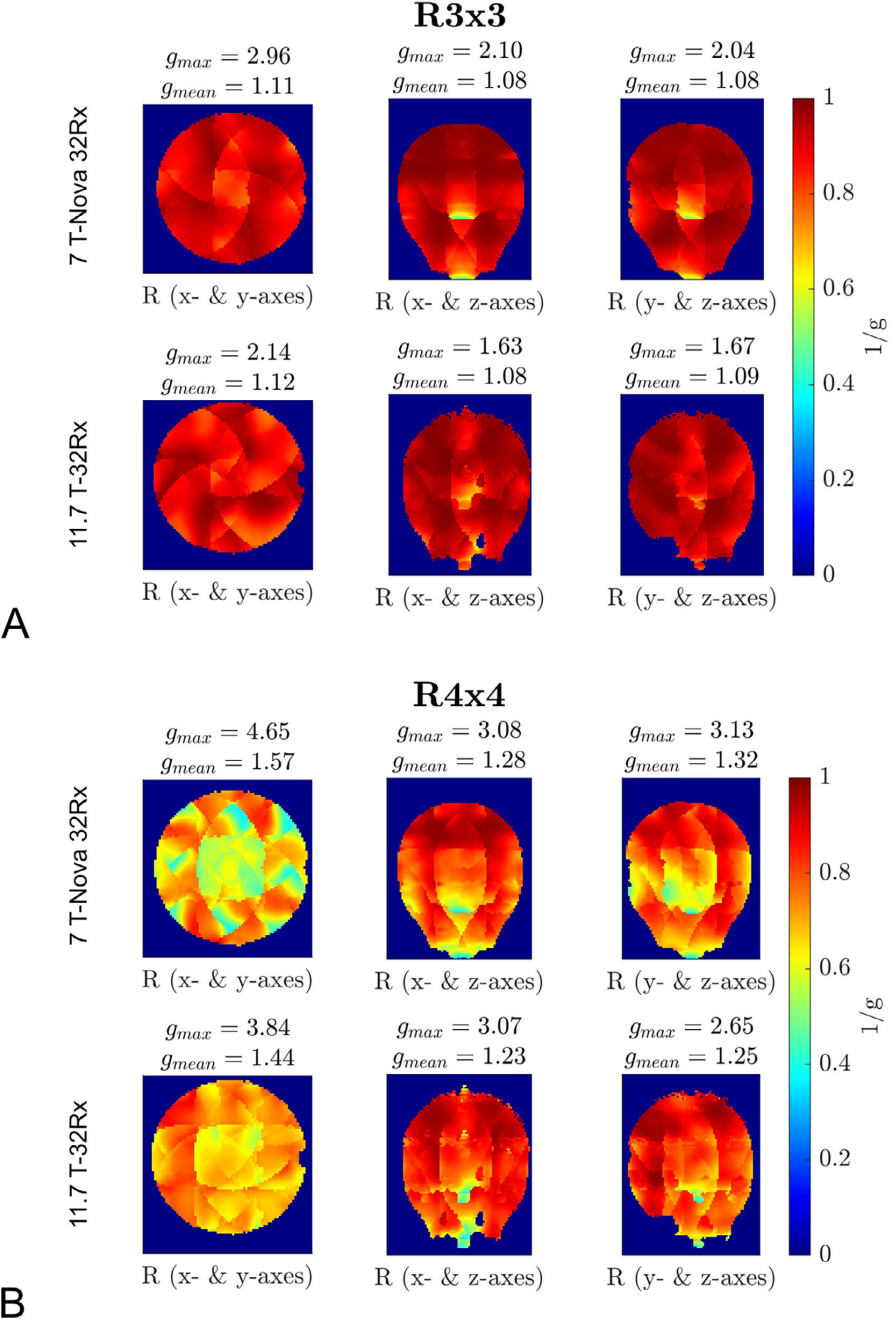
Comparison of measured inverse g‐factor maps for six 2D acceleration patterns in the lightbulb phantom at 7T and 11.7T. Maximum and mean g‐factor values are provided for each scenario.

Inverse g‐factor maps were computed from the experimentally characterized receive profiles and are illustrated in Figure 9. The maximum and average g‐factor values were calculated across the entire 3D volume of the lightbulb phantom. For all 3 × 3 accelerations, the maximum g‐factors decreased at 11.7T. However, there was no clear improvement in the mean g‐factors with increasing field strength. In contrast, for the 4 × 4 accelerations, both the maximum and mean g‐factor values improved with increasing field strengths. While electrodynamic calculations clearly indicate a decrease of the g‐factors for higher fields [21], the details of the coil design and layout may play the dominant role in the parallel imaging performance.

### 3.5 In Vivo Imaging

**FIGURE 10:**
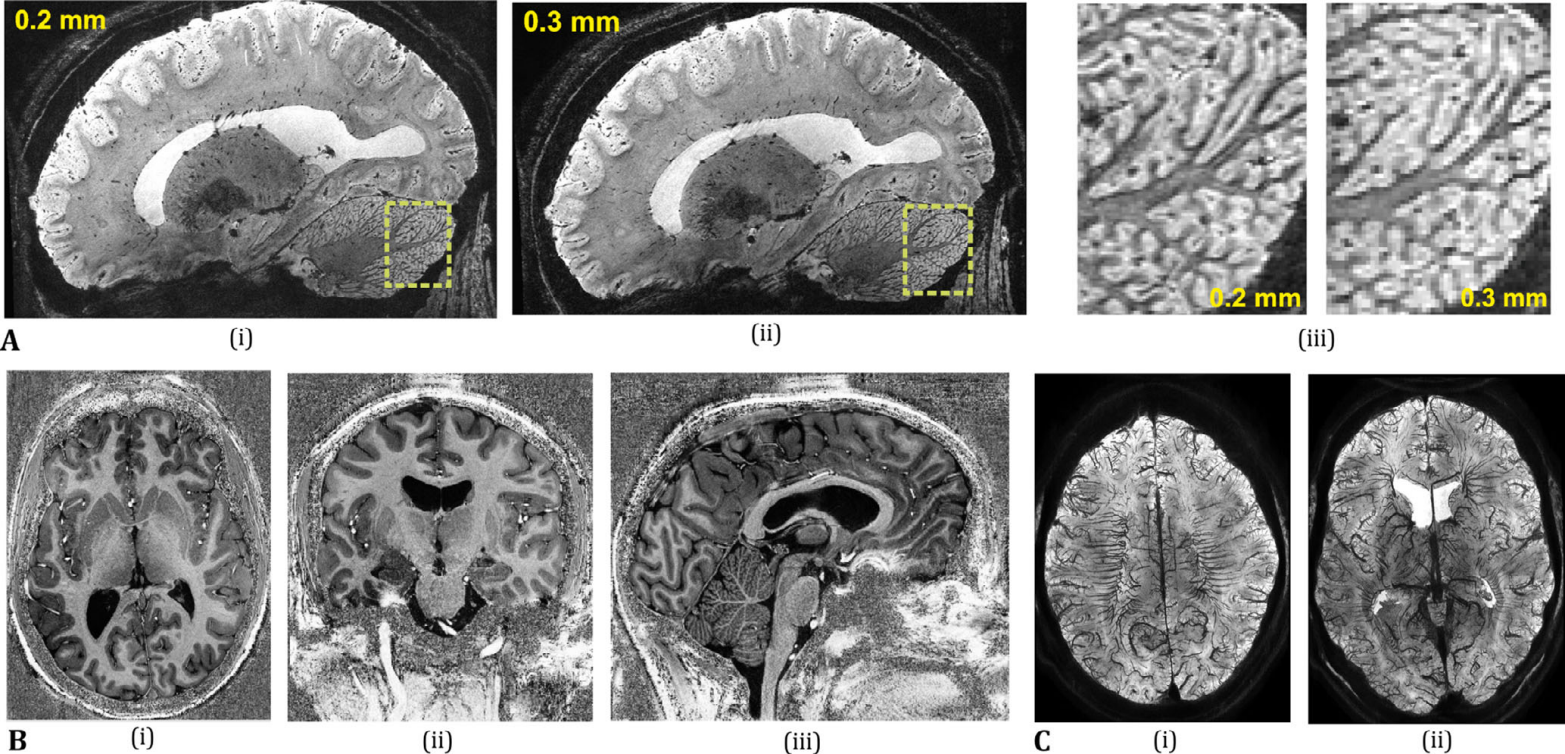
(A) 3D‐EPI images with isotropic resolution of 0.2 (i) and 0.3 mm (ii). The zoomed 0.2 mm image in (iii) demonstrates finer details. (B) MP2RAGE images demonstrating whole brain coverage and mitigation of the B_1_^+^ field inhomogeneity problem at 11.7T. (C) Minimum intensity projected images computed from the 0.2 mm 3D‐EPI acquisition.

In vivo images from one healthy volunteer scanned with the coil are shown in Figure 10. First row (A) shows 3D EPI images with 0.2 mm (i) and 0.3 mm (ii) isotropic resolution acquired in 25 and 5 min, respectively. While it is hard to appreciate the differences between the two whole brain images, the zoomed images (iii) highlight the different voxel sizes. Figure 10C shows minimum intensity projected (3 mm thickness) images from the 0.2 mm 3D‐EPI images, showing very fine venous structures.

The MP2RAGE images (B) were acquired with an isotropic resolution of 0.6 mm in about 10 min. These images demonstrate whole brain coverage at 11.7T with single‐row 8‐channel loop‐based transmit arrays and parallel transmission RF pulses mitigating the RF field inhomogeneity problem, with no severe B_1_^+^ artifact. B_0_ offset artifacts (hyperintensities) remain occasionally and are the subject of future work. The peak local SAR values for these scans are as follows: 3D‐EPI 0.2 mm—3.7 W/kg; 3D‐EPI 0.3 mm—4.7 W/kg and MP2RAGE—11.7 W/kg.

## 4 Discussion

We have presented the design, coil construction, and validation of an 8‐channel transmit 32‐channel receive 11.7T head coil and characterized both the transmit and receive performance of the constructed array. While head coils with up to 16 transmit and 128 receive channels have been developed for 10.5T scanners [17, 18], the presented coil design is unprecedented and the first of its kind for 11.7T MRI applications. Both the transmit and receive arrays were constructed using conventional loops, and our work demonstrates that excellent performance can be extracted with this design approach at 500 MHz. We examined SNR gain from 7T, the most used UHF platform, to 11.7T, which is currently the highest B_0_ field for human MRI.

The 11.7T receive array was constructed on a helmet, size‐matched with the reference industry standard 32‐channel 7T head coil, which was used as the reference coil in our study. It has been shown recently that the reference 7T head coil achieves slightly more than 80% of the uiSNR in the centre of the lightbulb phantom (figure S5 in Reference [18]). The 11.7T coil achieved a 3‐fold gain in the central SNR—a B_0_^2.14^ gain which agrees well with the uiSNR theory, albeit in a spherical phantom [20, 22, 24]. Hence, it can be concluded that the fully overlapped 32‐channel 11.7T receive‐only array has captured at least 80% of the uiSNR, because the SNR gain in the centre is in line with the theoretical prediction [45]. In addition, 10.5T versus 7T uiSNR calculations provided a 2.6‐fold increase in the centre of the lightbulb phantom [49]. Considering the B_0_ field dependence, the 3‐fold gain achieved by the 11.7T coil further confirms that the 11.7T coil performance is in line with the uiSNR calculations [49].

Our results demonstrate the critical importance, especially of the coil layout. Furthermore, decoupling strategies, circuitry, and the choice of materials play a role in extracting optimal coil performance at very high Larmor frequencies. Recent work [49] employing gapped/partially overlapped receive array design at 10.5T captured only 65% and 58% of uiSNR in simulation and experiment in the centre of the lightbulb phantom, respectively, whereas the same design was nearly optimum at 7T, capturing up to 90% of uiSNR. This led to the conclusion that a greater number of receive elements combined with larger transceiver elements is essential to capture the available SNR at > 10T [17, 49]. We have been able to establish with reasonable confidence that our 32‐channel array captured at least 80% of the uiSNR in the centre at 11.7T, and our results are more in agreement with Lattanzi and Sodickson [50], in which circular loops in principle capture 80% of the uiSNR at 11T.

Although the sample noise dominance is favorable at 10.5T compared to 7T for the smaller loops of the gapped design, it is possible that the uneven distribution of capacitors to implement self‐decoupling [17] compromises the performance of the coil severely at 10.5T, resulting in inferior performance as observed in both simulation and experiments. The individual loop design, coil layout, and the decoupling strategy of the presented 11.7T array are fundamentally different from the 31 and 63‐element 10.5T arrays considered by Waks et al. [17] and Zhang et al. [49].

In the thin periphery, however, uiSNR is expected to gain linearly with field strength [51]. We report an SNR gain of a factor of 2.65 in the peripheral 20 mm ROI, representing a B_0_^1.90^ gain with our 32‐channel array. It is important to note that the reference 7T coil achieved 16%–40% of the uiSNR at 5 to 20 mm distance from the phantom surface [18]. The supra‐linear peripheral SNR gain means that the 11.7T coil captures more of the uiSNR in the periphery compared to the reference 7T coil. We attribute this to the low‐loss circuit elements, decoupling strategy, and minimizing losses before the preamplifier.

To ensure that the measured SNR gain is inherent within the coil and not influenced by any external factors such as the optimized RF shield within which the receive array is placed, we measured the SNR of the 11.7T coil with and without the RF shield. There was no noticeable change in the receive performance of the array, especially in the centre of the phantom. These maps are shown in Figure S5.

The Iseult scanner is currently equipped with 8‐transmit and 32‐receive channels, and the constructed array was designed to match this configuration. Upgrading the scanner with 64 receive channels is planned for 2029. Future 11.7T receive arrays with > 32 elements will explore combining higher channel count receive arrays with complementary structures and/or transceivers to further enhance the central SNR because loop‐based receive arrays alone cannot capture all the available SNR at 11.7T [16, 49, 52, 53].

## 5 Conclusion

An 8‐channel transmit 32‐channel receive 11.7T head coil has been custom built. While the transmit array design was conceived and optimized using EM simulation tools combined with RF pulse designs, the receive array design was intuitive and empirical, with considerations for coil loading, decoupling, and minimizing losses before the preamplifier. The transmit array performance was validated using B_1_^+^‐field mapping and MR thermometry to establish safe operating limits to comply with regulatory requirements, while the receive array exhibits supra‐linear SNR gains in agreement with theoretical estimates. In vivo data demonstrates whole brain coverage and high‐resolution imaging capability.

## Funding

This work was supported by the European Union's Horizon 2020 research and innovation programme (885876). Agence Nationale de la Recherche (ANR) (ANR‐21‐ESRE‐0006).

## Conflicts of Interest

Son Chu is a part‐time employee at MR CoilTech Limited, Glasgow, UK, and Shajan Gunamony is a shareholder of MR CoilTech Limited.

## Supporting information


**Table S1:** List of components in the equivalent circuit diagram of a single receive element shown in Figure 3B. All fixed capacitors are 100BB series or equivalent from Kyocera AVX, USA and Dalian Dalicap Technology, China.
**Table S2:** Frequency‐dependent dielectric properties of GM and WM reported in the literature [4].
**Table S3:** Comparison of target and experimentally measured dielectric properties of the developed tissue‐equivalent phantom across MRI‐relevant frequencies.
**Figure S1:** Average projected dimensions of the grid lines on the surface of the helmet.
**Figure S2:** (A) Dielectric property measurement of the solution mixture using a commercial dielectric probe. (B) A 3D‐printed light‐bulb polycarbonate shell (C) Measured relative permittivity and conductivity as a function of frequency.
**Figure S3:** (Left) Histogram of the simulated and measured temperatures shown in Figure 7. (Right) Scatter plot of the temperature map shown in Figure 7.
**Figure S4:** Noise covariance matrix plotted for (A) 7T—Nova 32‐channel receive array (B) 11.7T—Home‐built 32‐channel receive array.
**Figure S5:** 8 × 8 S‐parameter matrices of the transmitter array shown for (A) simulation, (B) measurement without receive array, and (C) measurement with the actively detuned receive array.
**Figure S6:** Comparison of 1‐D signal intensity profiles extracted along three orthogonal lines passing through the centre of the lightbulb‐shaped phantom at 11.7T.

## Data Availability

The data that support the findings of this study are available from the corresponding author upon reasonable request.
