# Supplementary material for: Design of an 8‐Channel Transmit 32‐Channel Receive 11.7T Head Coil and Evaluation of SNR Gains
Source: Magn Reson Med. 2026 Jun 25;96(4):2004–18. doi: 10.1002/mrm.70473 (PMC13419043; doi:10.1002/mrm.70473)
Supplement: Supplementary file 1 — Table S1: List of components in the equivalent circuit diagram of a single receive element shown in Figure 3B. All fixed capacitors are 100BB series or equivalent from Kyocera AVX, USA and Dalian Dalicap Technology, China. Table S2: Frequency‐dependent dielectric properties of GM and WM reported in the literature [4]. Table S3: Comparison of target and experimentally measured dielectric properties of the developed tissue‐equivalent phantom across MRI‐relevant frequencies. Figure S1: Average projected dimensions of the grid lines on the surface of the helmet. Figure S2: (A) Dielectric property measurement of the solution mixture using a commercial dielectric probe. (B) A 3D‐printed light‐bulb polycarbonate shell (C) Measured relative permittivity and conductivity as a function of frequency. Figure S3: (Left) Histogram of the simulated and measured temperatures shown in Figure 7. (Right) Scatter plot of the temperature map shown in Figure 7. Figure S4: Noise covariance matrix plotted for (A) 7T—Nova 32‐channel receive array (B) 11.7T—Home‐built 32‐channel receive array. Figure S5: 8 × 8 S‐parameter matrices of the transmitter array shown for (A) simulation, (B) measurement without receive array, and (C) measurement with the actively detuned receive array. Figure S6: Comparison of 1‐D signal intensity profiles extracted along three orthogonal lines passing through the centre of the lightbulb‐shaped phantom at 11.7T. [file MRM-96-2004-s001.pdf]

Supplementary Information:

Design of an 8-Channel Transmit 32-Channel Receive 11.7T Head Coil and Evaluation of SNR Gains

1. Component description

Components	Values	Description
C_L	5.1pF (row 1); 3.6pF (row 2); 3pF (row 3 & 4)	Distributed capacitors in the loop; 100B series or equivalent fixed capacitors.
C_{M1}	= C_L	Impedance matching capacitor.
C_{M2}	Select by test	C_{M2} depends on the individual coil element loading.
C_B	= C_L	Active blocking capacitor.
C_S	5 to 10 pF	Series capacitor, adjusted for preamp decoupling.
C_g	390pF	DC block / bypass capacitor
RFC	390nH	RF choke (1008CS-102XGLC, Coilcraft, USA)
D_1, D_2	N/A	PIN diode for active detuning (MA4P7464F-1072T, MACOM Technology Solutions, USA)
D_{CR}	N/A	Cross diode for passive blocking (UMX9989AP, Microchip Technology, USA)
L_B	Depends on C_B	Hand-wound inductor for active detuning

Table S1: List of components in the equivalent circuit diagram of a single receive element shown in figure 3B. All fixed capacitors are 100BB series or equivalent from Kyocera AVX, USA and Dalian Dalicap Technology, China.

2. Grid dimensions

To help with coil construction, grid lines were drawn on the surface of the helmet. The average dimensions of the grid in each row is given in figure S1. The loops are built and overlapped by using the grid lines as reference. Elements in rows 2,3 and 4 overlaps with two of the upper row elements. The overlap distance beyond the grid line is approximately 12mm. In addition, each element overlaps with the two adjacent elements within each row. This is approximately 5mm. Note that there will be minor differences in the dimensions of the individual grid and the overlapped loops within each row due to shape of the helmet. The overlapped loop arrangement can be seen in Figure 3A.

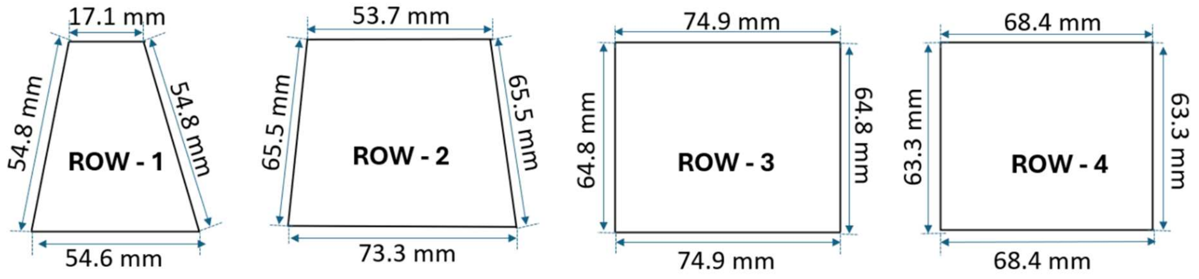

Figure S1. Average projected dimensions of the grid lines on the surface of the helmet.

3. Phantom Preparation

To quantify the SNR gain at 11.7T relative to 7 T, a tissue-equivalent phantom was developed to closely replicate the dielectric properties of human brain tissue at these frequencies, closely following the lightbulb phantom developed at CMRR, University of Minnesota [1]. The effective dielectric properties of human brain tissue were calculated as weighted averages of grey matter (GM) and white matter (WM) contributions, following standard mixture formulations:

$$\varepsilon_{eff} = f_{GM} \times \varepsilon_{GM} + f_{WM} \times \varepsilon_{WM} \quad (1)$$

$$\sigma_{eff} = f_{GM} \times \sigma_{GM} + f_{WM} \times \sigma_{WM} \quad (2)$$

where f_{GM} and f_{WM} represent the fractional volume contributions of GM and WM, respectively, and ε and σ denote relative permittivity and electrical conductivity. The fractional volume of GM and WM in human brain has been reported in the literature as 0.58 and 0.42, respectively [2], [3]. Frequency-dependent dielectric properties of GM and WM were derived from previously published datasets [4] and used to calculate the effective dielectric targets for phantom optimization.

Frequency (MHz)	Brain tissue	ε_r	σ (S/m)
297.18	GM	60.10	0.69
	WM	43.80	0.41
447	GM	56.60	0.76
	WM	41.50	0.46
499.415	GM	55.80	0.78
	WM	41.00	0.47

Table S2: Frequency-dependent dielectric properties of GM and WM reported in the literature [4].

Due to its nontoxic nature and excellent water solubility, PVP10 was selected as the primary solute for dielectric control, as it produces fewer extraneous spectral signals than alcohol or sucrose for reliable electromagnetic analysis. Sodium chloride was added to adjust the ionic conductivity of the solution [5]. The optimised formulation comprised 60.98% deionized water, 38.05% polyvinylpyrrolidone (PVP10, Sigma-Aldrich, USA), and 0.98% sodium chloride (NaCl, Sigma-Aldrich, USA) by weight.

Electromagnetic characterisation of the mixture solution for the light-bulb phantom was conducted using a commercial dielectric probe (DAK 12, Schmid & Partner Engineering AG, Switzerland) to measure ε_r and σ across the relevant ultrahigh-field MRI frequencies, as illustrated in Figure S2A. Measurements were conducted at room temperature at 297.18 MHz, 447 MHz, and 499.415 MHz (Table S2).

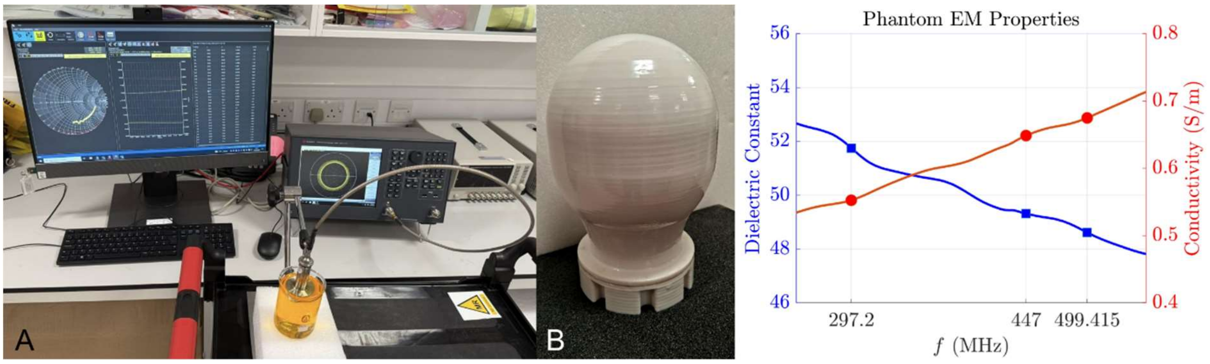

Figure S2: (A) Dielectric property measurement of the solution mixture using a commercial dielectric probe. (B) A 3D-printed light-bulb polycarbonate shell (C) Measured relative permittivity and conductivity as a function of frequency.

The mixture was then encapsulated in a lightbulb phantom, as shown in Figure S2B. Comparison between the target and measured dielectric properties is shown in Table S3, while the measured dielectric properties of the phantom are graphically depicted in Figure S2C.

Frequency (MHz)	Target Values		Measured Values	
	ε_r	σ (S/m)	ε_r	σ (S/m)
297.18	53.25	0.574	51.76	0.552
447	50.26	0.632	49.32	0.648
499.415	49.58	0.651	48.62	0.674

Table S3: Comparison of target and experimentally measured dielectric properties of the developed tissue-equivalent phantom across MRI-relevant frequencies

The measured dielectric properties closely matched the calculated target values across all frequencies, confirming the fidelity of the phantom in replicating the electromagnetic behaviour of average human brain tissue.

4. MR Thermometry

With reference to the temperature maps presented in Figure 7 of the main article, the corresponding histogram and scatter plot of the measured and simulated temperatures are provided in Figure S3. The scatter plot was generated following interpolation. The root mean square error (RMSE) between the measured and simulated temperatures is 0.07 °C. The mean temperature measured across the phantom using thermometry is 0.16 °C, with a maximum value of 0.54 °C. The larger mean value in the experimental data is believed to be due to an unaccounted residual field drift.

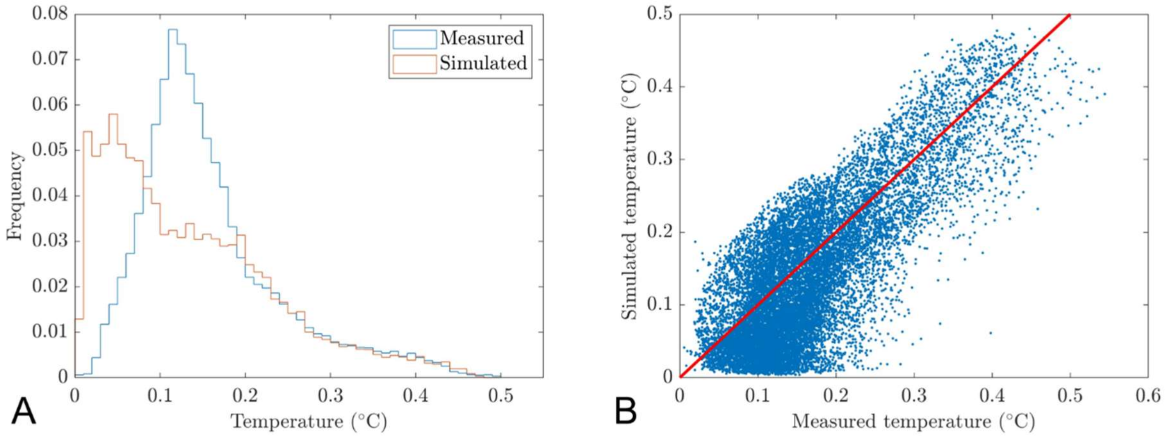

Figure S3: (left) Histogram of the simulated and measured temperatures shown in figure 7. (right) Scatter plot pf the temperature map shown in figure 7.

5. Noise Covariance

Figure S4 shows the noise covariance matrices, computed after stacking all k-space points with zero voltage in one single vector for each coil element, for the 7T Nova 32RX coil (A) and the home-built 11.7T 32Rx coil (B). The 11.7T array showed reduced noise level as the Frobenius norm of the covariance matrix was 4.1394×10^{-5} for the 7T coil compared to 3.3524×10^{-5} for the 11.7T coil, which corresponds to 23% higher noise for the 7T coil.

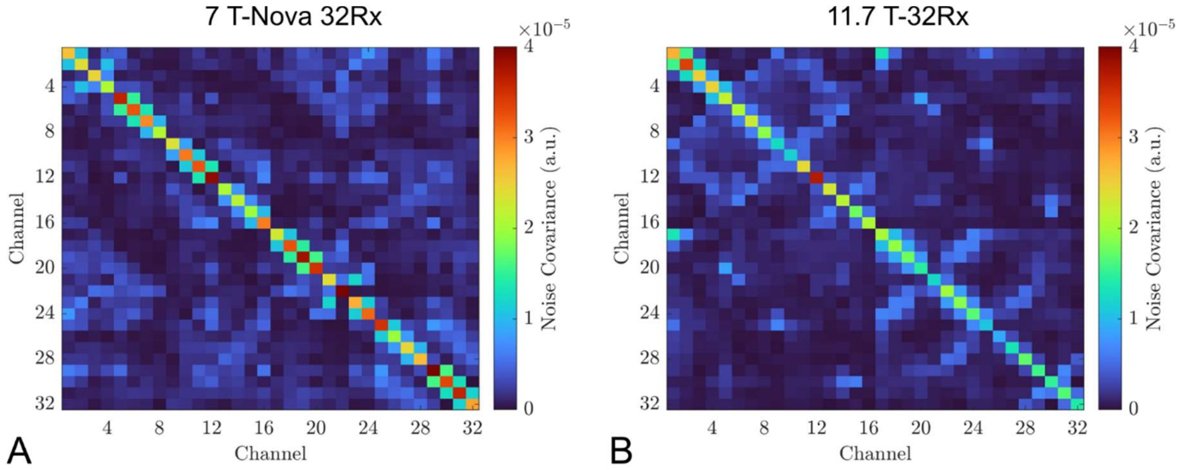

Figure S4: Noise covariance matrix plotted for (A) 7T - Nova 32-channel receive array (B) 11.7T – Home-built 32-channel receive array.

6. S-parameter matrix

The simulated and measured S-matrix of the transmit array is shown in Figure S5. The measured values shown in B and C are without the receive array and with the actively detuned receive array within the transmit array, respectively. Only the tune and match of the transmit elements are adjusted after inserting the receive array. Although there is a mismatch in the absolute value of the S-parameters, we consider obtaining clean S_{ii} responses with the completed array over a span of at least 10%, as displayed in Figure 4, an important qualitative measure rather than quantitatively matching the S-parameter values at a single frequency. However, prior work has adapted the coil model to match the measured S-matrix. In our case, the mismatches are accounted

for in the calibration matrix explained in Section 2.4. The average value of all the off-diagonal elements in the simulated model is -18.97 dB, whereas it is -23.30 dB for the transmit array and -23.20 dB for the transmit array with the actively detuned receive array.

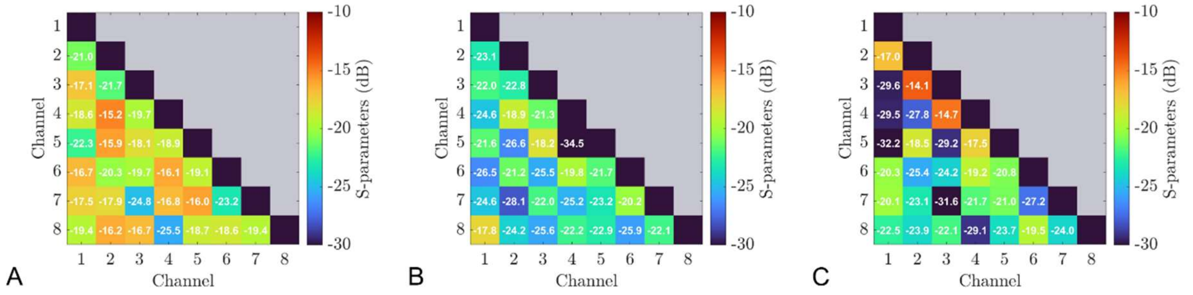

Figure S5: 8 × 8 S-parameter matrices of the transmitter array shown for (A) simulation, (B) measurement without receive array, and (C) measurement with the actively detuned receive array.

7. SNR Measurements with and without RF Shield:

To ensure that the observed SNR gain is intrinsic to the constructed 32-channel array and not influenced by external factors such as the presence of the receive array within the optimised RF shield, SNR measurements at 11.7 T were evaluated along three orthogonal directions through the centre of the lightbulb-shaped phantom.

Data were acquired without the RF shield and with the folded-end RF shield, for which two repeated measurements (Measurement 1 and Measurement 2) were performed to assess consistency and reproducibility. The 1-D profiles in Figure S6 show comparable SNR at the centre of the phantom, with minor differences attributed to slight positional variations between the setups.

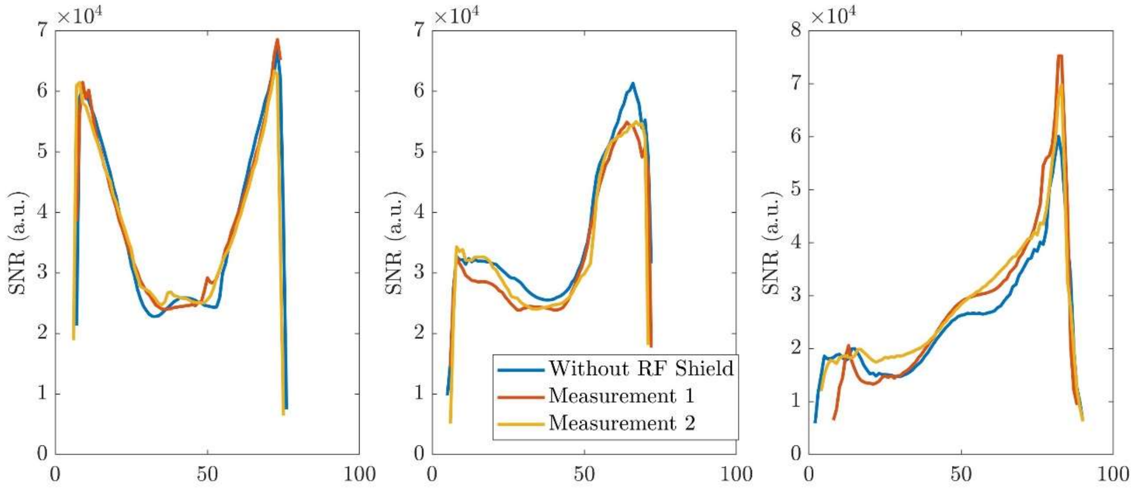

Figure S6: Comparison of 1-D signal intensity profiles extracted along three orthogonal lines passing through the centre of the lightbulb-shaped phantom at 11.7 T

References

[1] R. Lagore et al., “A 128-channel receive array with enhanced signal-to-noise ratio performance for 10.5 T brain imaging,” *Magnetic Resonance in Medicine,* vol. 93, no. 6, pp. 2680-2698, 2025.

[2] A. Irimia, “Cross-sectional volumes and trajectories of the human brain, gray matter, white matter and cerebrospinal fluid in 9473 typically aging adults,” *Neuroinformatics,* vol. 19, no. 2, pp. 347-366, 2021.

[3] R. Gur, B. Turetsky, M. Matsui, M. Yan, W. Bilker, P. Hughett and R. Gur, “Sex differences in brain gray and white matter in healthy young adults: correlations with cognitive performance,” *Journal of Neuroscience,* vol. 19, no. 10, pp. 4065-4072, 1999.

[4] C. Gabriel, “Compilation of the dielectric properties of body tissues at RF and microwave frequencies,” No. ALOETR19960037, 1996.

[5] C. Ianniello, J. de Zwart, Q. Duan, C. Deniz, L. Alon, J. Lee, R. Lattanzi and R. and Brown, “Synthesized tissue-equivalent dielectric phantoms using salt and polyvinylpyrrolidone solutions,” *Magnetic Resonance in Medicine,* vol. 80, no. 1, pp. 413-419, 2018.
